# Structural Characterization of Lignocresols from Transgenic and Wild-Type Switchgrass

**DOI:** 10.3390/polym10070727

**Published:** 2018-07-02

**Authors:** Hao Ren, Wenyuan Tian, Fan Shu, Dongliang Xu, Chunxiang Fu, Huamin Zhai

**Affiliations:** 1 Jiangsu Co-Innovation Center for Efficient Processing and Utilization of Forest Resources, Jiangsu Provincial Key Lab of Pulp and Paper Science and Technology, Nanjing Forestry University, No.159, Longpan Rd., Xuanwu District, Nanjing 210037, China; renhao@njfu.edu.cn (H.R.); wy_tian0224@163.com (W.T.); shufan2326@126.com (F.S.); xdl1218@126.com (D.X.); 2 Qingdao Institute of Bioenergy and Bioprocess Technology, Chinese Academy of Science, Qingdao 266101, China; fucx@qibebt.ac.cn

**Keywords:** switchgrass, lignocresols, transgenic sample, structural characterization

## Abstract

Cafferic acid-*O*-methyltransferases (COMT) down-regulated transgenic and wild-type switchgrass were separated into lignocresols (LCs) and sugars by a phase separation method involving 72% sulfuric acid and cresol. The isolated LCs were characterized by FTIR, GPC, ^1^H NMR and 2D-HSQC to understand potential structural modification caused by transgenic engineering lignin or phase separation treatment. No significant changes were found in terms of molecular weights and the amount of incorporated *p*-cresols between transgenic and wild-type switchgrass LCs. However, the compositions, ratios of syringyl (S) units to guaiacyl (G) units, were changed significantly leading to decrease in S units and increase in G units for transgenic switchgrass LC. The benzodioxane structures and 5-hydroxyguaiacyl units were observed in the 2D-HSQC implied that 5-hydroxyconiferyl alcohol was incorporated into lignin as a result of COMT-down-regulation in the transgenic process.

## 1. Introduction

In recent years, because of the rapid growth of the global population, rapid economic development, and improved standards of living, the demand for energy has increased dramatically worldwide [1]. The use of fossil fuels increases the emission of harmful substances such as SO_2_, CO_2_, and dust, leading to exacerbated environmental pollution and global climate change [2]. How to overcome the energy shortage and environmental pollution has become a great challenge for the present and future. Therefore, renewable and clean energy has become the focus of attention. Plant-based bioenergy has the advantages of less environmental pollution, renewable nature, potential low-cost, and wide applications [3], being considered as an ideal choice for the future energy generation [4]. In the process of cellulosic ethanol production, cellulose and polysaccharides are hydrolyzed to fermentable sugars, whereas lignins are left over as insoluble residues that have not been utilized efficiently in a significant scale due to their complex structures.

Switchgrass (*Panicun virgatum* L.) is a dedicated energy crop identified by the U.S. department of energy [5]. Fu et al. reported that down-regulation of the switchgrass cafferic acid 3-*O*-methyltransferase (COMT) gene modestly decreases lignin content, reduces the syringyl:guaiacyl lignin monomer ratio, improves forage quality, and increases ethanol yield by up to 38% using the conventional biomass fermentation processes. The down-regulated lines require less severe pretreatment and 300–400% lower cellulase dosages for equivalent product yields using simultaneous saccharification and fermentation with yeast [6].

To realize the comprehensive and economic industrial-scale production of biomass energy, high value-added utilization of lignin has been long focused. However, when considering the application of lignins, it is very important to track the changes in lignin structure during the transgenic process. In addition, some bits of important structures would be lost during the acidic or alkaline or ball milling preparation process. In this study, COMT regulated switchgrass was compared to its wild-type with respect to the structural characterization of lignocresols (LCs) after being subjected to a high acidic phase separation method [7]. Benzodioxane structures and 5-hydroxyguaiacyl units which were formed during the transgenic process were proved to be stable under the phase separation conditions and their characteristic C–H correlated signals in 2D-HSQC NMR spectra were identified distinctly. This work demonstrated again that phase separation is an effective method for separating lignins from carbohydrate in a biorefinery concept. The separated lignin derivatives, LCs, maintained most of their β-ether linkages and had high hydroxyl content due to the incorporation of cresol.

## 2. Materials and Methods

### 2.1. Materials

*p*-Cresol (chemical pure, CP), 72% sulfuric acid (CP), solid KBr in solid (analytical reagent, AR grade), tetrahydrofuran (THF), acetone, and diethyl-ether (CP) were purchased from Sinopharm Chemical Reagent Co. (Shanghai, China). The 60-mesh pass powder of COMT down-regulated switchgrass (transgenic) and wild-type switchgrass (control) were provided by Dr. Fu Chunxiang of the Qingdao Institute of Bioenergy and Bioprocess Technology, Chinese Academy of Sciences. The chemical components of transgenic and control samples were as follows: moisture, 10.36% and 10.12%; Klason lignin, 17.21% and 20.56%; acid soluble lignin, 1.96% and 1.98%; cold water extractives, 10.39% and 10.93%; hot water extractives, 15.19% and 16.37%; 1% NaOH extractives, 33.37% and 32.29%; and ash, 1.72% and 1.68%, respectively.

### 2.2. Phase Separation Procedure

**Figure 1:**
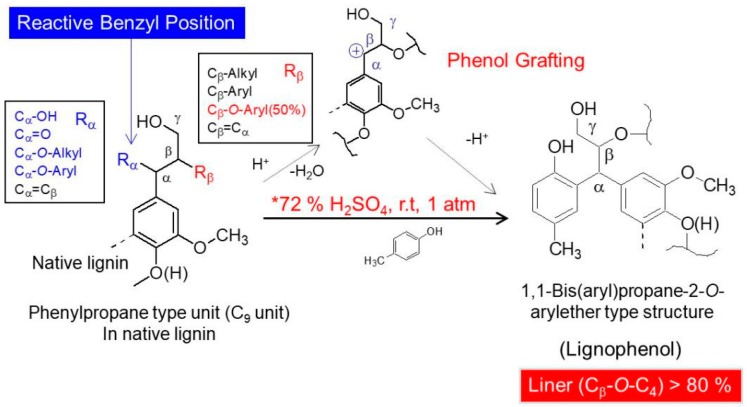
A schematic model of phase separation system [8].

*p*-Cresol (10 mL/g biomass) was added to switchgrass meals with stirring. After 10 min, 72% sulfuric acid (20 mL/g biomass) was added to the mixture and the vigorous stirring was continued at room temperature for the prescribed time. The resultant mixture was let stand for 10 min. The separated cresol phase was added drop-wise to an excess (200 mL) of ethyl ether with vigorous stirring. The precipitates were collected and dissolved in acetone (80 mL), and insoluble materials were removed by centrifugation. The acetone solution was concentrated to 10 mL under reduced pressure and added drop-wise to an excess (200 mL) of ethyl ether with stirring. The precipitated lignin derivative (LCs, ether-insoluble) was collected by centrifugation and dried over P_2_O_5_ after evaporating the solvent. The ether soluble fractions were not accounted into the yield of LCs. The schematic model of phase separation is shown in Figure 1.

### 2.3. Characterization of LCs

The FT-IR spectra of LCs were obtained on a Bruker VERTEX80 Spectroscope (Bruker, Berlin, Germany) using KBr discs. The spectra were recorded in the range from 400 cm^−1^ to 4000 cm^−1^ with a resolution of 4 cm^−1^ over 32 scans. Calibration for the weight-average molecular weight (*M*_w_), number-average molecular weight (*M*_n_), polydispersity (*M*_w_/*M*_n_), and the gel permeation chromatography (GPC) of lignins were determined on a PL-GPC50 plus Integrated GPC System (Varian Inc., Nagoya, Japan) equipped with a Waters 2410 RID detector. Sepax Mono-GPC columns (100 Å, 300 Å, 500 Å, 10 mm ID × 300 mm) were connected in a series and tetrahydrofunan (THF) was used as an eluent under the flow rate of 1.0 mL/min. A series of polystyrene (Mw: 200,000, 100,000, 500,000, 30,000, 20,000, 10,000, 4300, 2000, 108) was used as standard to make calibration line.

^1^H NMR spectra of LCs in C_5_D_5_N-CDCl_3_ (1:3, *v*/*v*) and acetylated LCs in CDCl_3_ were recorded on a 300 MHz NMR spectrometer. *p*-Nitrobenzaldehyde was used as the internal reference for the measurements. The amounts of incorporated cresol were calculated based on the signal intensity of its methyl protons (1.6–2.4 ppm) against aromatic protons (7.8–8.4 ppm) of internal standard (*p*-nitrobenzaldehyde) on ^1^H NMR spectra of original lignocresols. The hydroxyl group contents were determined from acetyl proton signals on ^1^H NMR spectra of acetylated lignocresols. The cresolic methyl proton signals overlapped in the region of acetyl protons were estimated based on the relative intensity of cresol methyl to aromatic proton signals in the spectra of original lignocresols [9]. The methoxy content were calculated according to the methods reported by Aberu and Freire (1995) [10,11].

Elemental analysis was performed on the lignin samples using a Perkin Elmer 2400 II Elemental Analyser instrument (PerkinElmer, Shanghai, China). In preparing the samples for analysis, first they were dried at 40 °C overnight by vacuum drying oven to remove any moisture. To measure carbon, hydrogen, nitrogen and sulfur contents, 1–2 mg samples were encapsulated in a tin container. The analysis results were obtained via gas chromatography, and compared with those of standard materials.

2D-HSQC NMR spectra were recorded on a 600 MHz NMR spectrometer (Avance III, Bruker, Bern, Switzerland) equipped with a cryogenically-cooled 5 mm TCI gradient probe with inverse geometry at 25 °C [12]. A portion of each lignin sample (60 mg) was dissolved in DMSO-d6 (0.6 mL) as the deuterated NMR solvent. The central solvent peak was used as an internal reference (δC/δH 39.5/2.50). The HSQC experiments were obtained using Bruker’s “hsqcetgpsp.2” adiabatic pulse program with spectral widths from 0 to 12 ppm (9615 Hz) and from 0 to 165 ppm (24,900 Hz) for the ^1^H and ^13^C dimensions, respectively. The number of collected complex points was 2048 for the ^1^H dimension with a recycle delay (d1) of 1.5 s, 64 transients for the HSQC spectra and 256 time increments in the ^13^C dimension resulting in an overall experiment time of 18 h [13]. A 1JC-H value of 145 Hz was employed. Prior to Fourier transformation, the data matrices were zero-filled to 1024 points in the ^13^C dimension. A semiquantitative analysis of the intensity of the HSQC signals was then performed using Bruker’s Topspin 2.1 processing software, and the integral method reported by Rio et al. was employed [14].

## 3. Results and Discussions

### 3.1. Yields of LCs from Switchgrass through Phase Separation

The yields of LCs from transgenic and control sample are shown in Table 1. Because the cell wall of herbaceous plants was relatively bulky compared to woody plants, several reaction times (10 min, 20 min, 30 min, and 60 min) were selected to produce LCs from the transgenic and wild type samples. Both samples showed the maximum yield when the treatment time was 30 min in phase separation and the yields of LCs from transgenic and control switchgrass were 71.0% and 57.4%, respectively. This indicated that the yield of LCs was much higher than that from MWL, although it was lower than the LCs yields from woody plants [11]. This might be attributed to the loose cell wall structure of herbaceous plants and a lower degree of lignification compared to wood [15,16,17]. During the phase separation process, the lignin skeleton was selectively cleaved, resulting in many relatively small molecular lignin products [7,8]. After centrifugation, the LCs were collected in the organic phase, and part of the small molecule products was thought to be lost during the following purification process using diethyl ether and acetone. In Table 1, it can be observed that the yield increased gradually with the extension of reaction time from 10 min to 30 min, and slightly decreased after 30 min. To explain this phenomenon, it was considered that, at the beginning, as the time of phase separation increased, the frequency of cresol introduced increased gradually, and the yield of LCs increased. After 30 min, the obtained LCs would further degrade under acidic conditions leading to an increase in the molecular components lost during the subsequent refining process using organic solvents. In summary, for the transgenic and control switchgrass, 30 min was a good reaction time for phase separation.

### 3.2. FTIR Spectra of Switchgrass LCs

**Figure 2:**
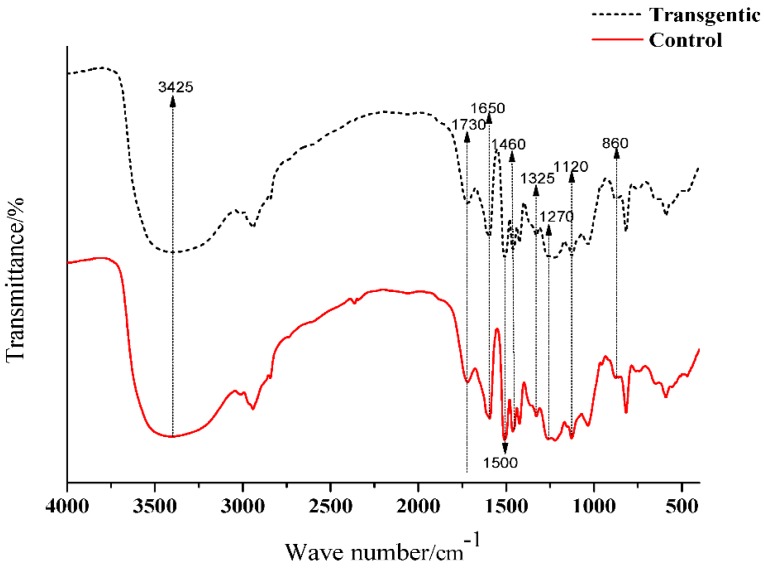
FTIR spectra of switchgrass LCs.

The FTIR spectra of LCs from transgenic and control switchgrass are shown in Figure 2. The peaks derived from syringyl propane units in the lignin skeleton were at 1325, 1220, and 1130 cm^−1^, and the peaks derived from guaiacyl propane units were clearly visible at 1270 and 1040 cm^−1^. In addition, there was a larger absorption peak at 3425 cm^−1^ derived from hydroxyl groups, at 1730 cm^−1^ from ester bond on the γ-position, and at 815 cm^−1^ there was an absorption peak due to the angular vibration of ortho-hydrogen on the benzene ring of the introduced *p*-cresol [15]. In the FTIR spectra, the structural characteristics of transgenic and control switchgrass were not significantly different, which also showed no significant difference compared to LCs from other herbaceous plants such as bamboo [16]. This suggested that the lignin units in switchgrass were the same as those in other herbaceous plants. In the transgenic plant, the numbers and ratios of lignin basic structural units (GSH units) may be changed, but lignin skeleton structure had not been changed. 

### 3.3. Molecular Weights of LCs and Amounts of Incorporated p-Cresols 

**Figure 3:**
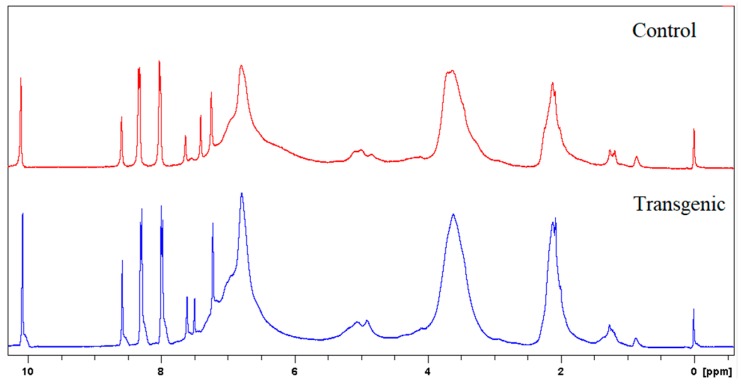
^1^H NMR spectra of LCs.

**Figure 4:**
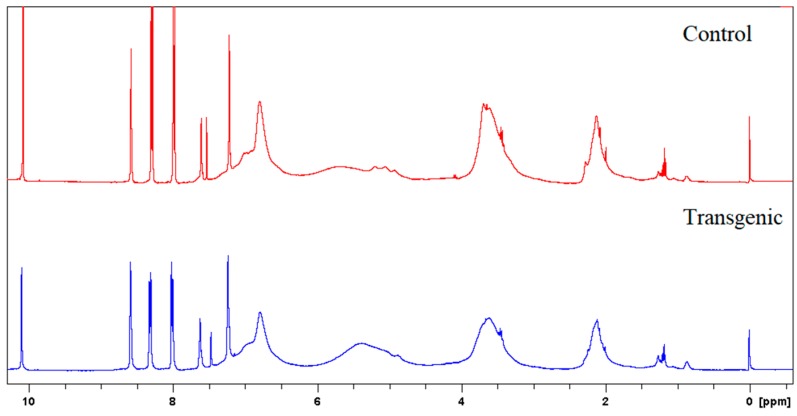
^1^H NMR spectra of acetylated LCs.

The molecular weights, amounts of combined cresols, and hydroxyl groups of LCs are shown in Table 2. There was no significant difference in the molecular weight and the ratio of distribution between transgenic and control switchgrass. The polydispersity of the molecular weights of LCs were all close to 1, indicating that the size of the molecular chain was more uniform. After acetylation, the molecular weights of both samples increased significantly, because the amounts of hydroxyl groups (calculated based on Figure 3 and Figure 4) are high in LCs. The amounts of introduced *p*-cresols on Cα in both samples were ca. 0.8 mol/C_9_, which was consistent with the data reported for other kinds of herbaceous materials, and higher than the average level (0.7 mol/C_9_) in softwood [7,9]. This again indicated that the structure of herbaceous plants was relatively loose, and that under the phase separation conditions, the reaction accessibilities and reactivity were superior to those of woody plants [16,17].

### 3.4. Elemental Analysis and Methoxyl Content

The elemental analysis results for the lignins are shown in Table 3. The data show that the carbon and hydrogen contents increase from control to transgenic switchgrass. In the transgenic sample, the nitrogen content is higher than that in the control sample, whereas the sulfur content is lower. Nitrogen may be originated from the enzyme during the transgenic treatment process and sulfur may be originating from the residual sulfuric acid during phase separation. In lignin chemistry, the empirical formula of the macromolecule is commonly presented as a hypothetical hydroxyphenyl structural unit. This is known as the C_9_ formula, with six carbon atoms in the benzene ring plus three carbon atoms making up the propyl side-chain. The results are shown in Table 3. The methoxyl group contents decreased from the control to transgenic samples. In the transgenic sample, the number of methoxyl groups is close to 1 per C_9_ formula, indicating that the syringyl units had largely been decreased by the COMT down-regulation. This is due to the decreased activity of COMT enzyme that is responsible for methylating 5-hydroxyconiferaldyhye (leading to sinapyl alcohol) eventually to produce syringyl lignin units. As the results the acumination of 5-hydroxyconiferyl units (forming benzodioxane structures) resulted in decrease of S units, hence, the S/G ratio decreased [6,18,19].

### 3.5. 2D-HSQC NMR Spectra of LCs from Transgenic and Control Switchgrass

**Figure 5:**
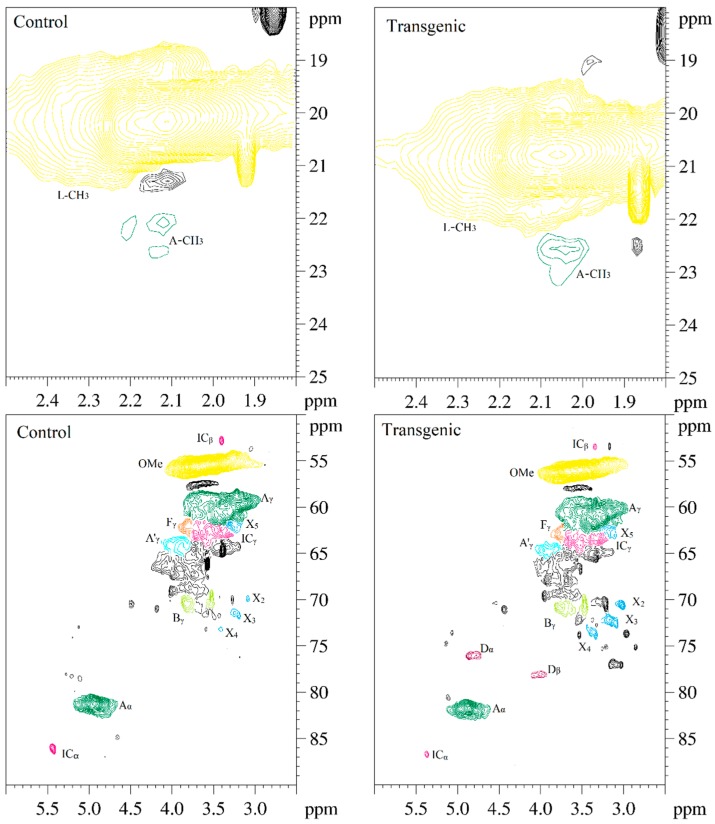

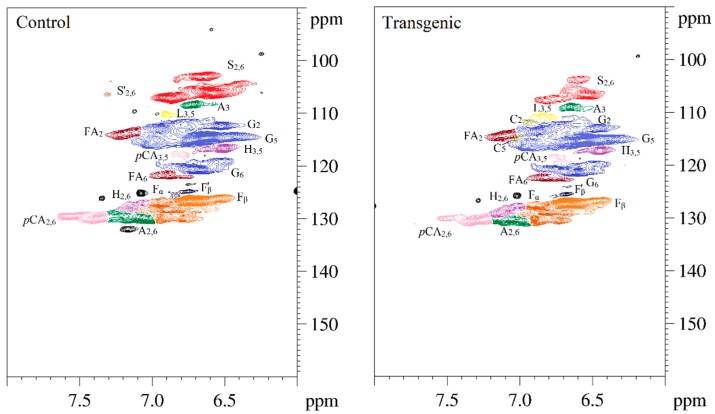
The 2D-HSQC NMR spectra of LCs from transgenic and control switchgrass.

**Figure 6:**
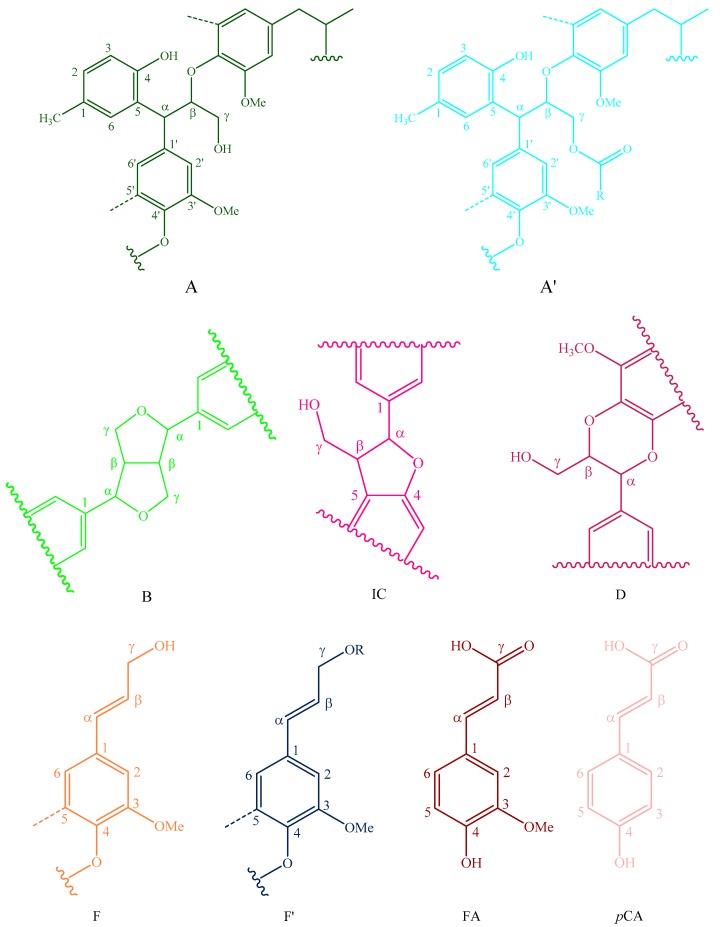

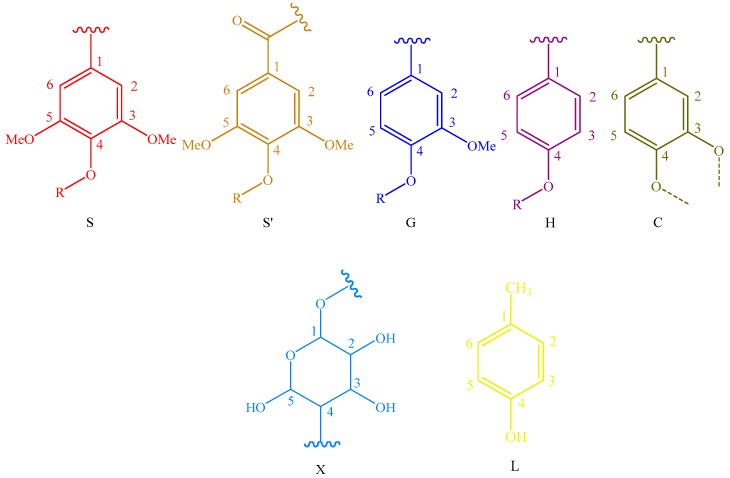
Main classical structures in the lignin preparations: (**A**) lignocresol; (**A’**) β-*O*-4 alkyl-aryl ethers with acylated γ-OH with *p*-coumaric acid; (**B**) resinol structures formed by β-β/α-*O*-γ/γ-*O*-α linkages; (**IC**) phenylcoumarane structures formed by β-5/α-*O*-4 linkages; (**D**) benzodioxane substructures; (**F**) *p*-hydroxycinnamyl alcohol end-groups; (**F’**) *p*-hydroxycinnamyl alcohol end-groups acylated at the γ-OH; (**FA**) ferulates; (*p*CA)p-coumarates; (**S**) syringyl units; (**S’**) oxidized syringyl units bearing a carbonyl at Cα; (**G**) guaiacyl units; (**H**) *p*-hydroxyphenyl units; (**C**) catechyl units; (**X**) xylopyranoside; and (**L**) *p*-cresol.

The side-chain regions (δC/δH 18–25/1.8–2.5, δC/δH 50–90/2.5–6.0) and aromatic ring region (δC/δH 90–160/6.0–8.0) in the 2D-HSQC NMR spectra of LCs from transgenic and control switchgrass are shown in Figure 5, and the relevant signal attribution is shown in Table 4. The relative quantitative method was used for determination of various structural units, as shown in Table 5, and the molecular structure of the main basic units is shown in Figure 6. Wild-type switchgrass has guaiacyl units (G, 48.42%), syringyl units (S, 41.22%), and *p*-hydroxybenzene units (H, 10.36%) related signals in the aromatic ring region. The S/G ratio is 0.85. The transgenic switchgrass also has signals related to guaiacyl units (G, 69.46%), syringyl units (S, 19.70%) and *p*-hydroxybenzene units (H, 10.84%) in the aromatic ring region, and the S/G ratio is 0.28. These results indicate that lignins from transgenic and control switchgrass are typical GSH lignins, and that the S unit of switchgrass was decreased significantly in the transgene. The β-*O*-4 alkyl-aryl ethers in lignocresol (A) produced from original β-*O*-4 alkyl-aryl ethers (A) and those with acylated γ-OH with *p*-coumaric acid (A’) were observed in the side-chain region of both transgenic and control switchgrass. It is interesting to see that some phenylcoumarane structures (IC) and resinol structures (B) survived such an acidic treatment and the other typical structures were also observed in the side-chain region of both transgenic and control switchgrass. The relative ratios of β-*O*-4, β-5, and β-β linkages in transgenic switchgrass LCs were 74.51%, 19.34% and 6.14%, and those in control switchgrass LCs were 80.72%, 14.56% and 4.68%, respectively. Upon comparison, the β-5 structure was found to be higher in transgenic switchgrass LCs than that in control LCs whereas the β-*O*-4 structure was found to be lower, which is consistent with their composition results. In addition, related signals of precursor structures such as *p*-hydroxycinnamyl alcohol end-groups (F) and its acylated moiety (F’), ferulates (FA) and *p*-coumarates (*p*CA) were also observed. 

In general, COMT down-regulated transgenic switchgrass lignin demonstrated a decrease in the syringyl (S):guaiacyl (G) ratio and the *p*-coumarate:ferulate ratio (Table 5). The amount of total free phenolic OH groups in transgenic LCs was comparable with that in control samples, along with benzodioxane units (D_α,β_ signals in Table 4) or catechyl units (C_2,6_ signals in Table 4) formed in the transgenic sample. Furthermore, COMT down-regulation did not significantly change lignin’s molecular weights. These results are consistent with the data reported previously [20]. Although the native lignin structures had been changed during the phase separation, the semi-quantified compositions and interunit linkage distributions in LCs were basically representative of those before treatment, which allows them to be used to assess the changes of GSH units during the transgenic treatment.

## 4. Conclusions

Phase separation method was demonstrated to have some advantages when applying to fractionate switchgrass biomass into carbohydrate and lignin derivatives, LCs. Although native lignin structures (mainly α-hydroxyl groups of β-aryl ethers) were greatly modified in the phase separation process, almost all interunit linkages and lignin structural units remained in the products LCs. Although the molecular weight of LCs from transgenic sample was close to that of LC from the control, semi-quantified results from HSQC analysis indicated that COMT down-regulated transgenic switchgrass has less S units (lower S/G ratios) compared to the control sample. Benzodioxane structures formed from 5-hydroxyconiferyl alcohol in transgenic samples survived the phase separation. Due to the incorporation of cresol into LCs during the phase treatment, the isolated LCs contained high amount of hydroxyl group (mainly phenolic) implying that these LCs could be used for many applications.

## Figures and Tables

**Table 1:** Yields of lignocresols from transgenic and control lignin sample.

Sample	Yield % (Based on Klason Lignin)			
(Reaction Time)	10 min	20 min	30 min	60 min
Transgenic	53.75 ± 0.12	56.89 ± 0.13	71.01 ± 0.12	54.85 ± 0.15
Control	44.46 ± 0.15	44.89 ± 0.10	57.39 ± 0.13	43.87 ± 0.14

**Table 2:** Molecular weight and functional groups of LCs.

Sample	*M*_w_	*M*_n_	*M*_w_/*M*_n_	Combined Cresol (mol/C_9_)	PhOH (mol/C_9_)	AliOH (mol/C_9_)
Transgenic	3547 ± 23	2087 ± 28	1.70	0.84	1.12	1.06
Control	2341 ± 36	1636 ± 53	1.43	0.85	1.20	1.13
Acetylated-Transgenic	3444 ± 42	2464 ± 37	1.40	-	-	-
Acetylated-Control	4354 ± 45	2822 ± 32	1.54	-	-	-

**Table 3:** Chemical composition of switchgrass LCs.

	Analytical Composition (%)						Empirical Formula
	C	H	O	N	S	OCH_3_	
Transgenic	66.76	6.29	28.83	2.75	0.37	18.10	C_9_H_8.22_O_2.2_N_0.35_S_0.02_(OCH_3_)_1.05_
Control	62.92	5.97	28.45	2.27	1.39	19.58	C_9_H_7.97_O_2.1_N_0.32_S_0.08_(OCH_3_)_1.23_

**Table 4:** The 2D-HSQC NMR attribution of switchgrass LCs.

Lable	δ_C_/δ_H_ (ppm) ^a^	δ_C_/δ_H_ (ppm) ^b^	Assignment
A-CH_3_	22.02/2.12	22.5/2.05	C−H in lignocresol-CH_3_ (A)
IC_β_	52.75/3.40	53.45/3.34	C_β_−H_β_ in phenylcoumaran substructures (IC)
OMe	55.54/3.67	56.07/3.59	C–H in methoxyls (OMe)
A_γ_	61.07/3.42	61.73/3.36	C_γ_−H_γ_ in β–*O*–4 substructure of lignocresol (A)
A’_γ_	64.24/4.03	64.63/3.96	C_γ_−H_γ_ in γ-hydroxylated β–*O*–4 substructures (A’)
F_γ_	62.05/3.81	62.91/3.78	C_γ_−H_γ_ in *p*-hydroxycinnamyl alcohol (F)
IC_γ_	62.62/3.57	63.26/3.65	C_γ_−H_γ_ in phenylcoumaran substructures (IC)
B_γ_	70.29/3.83	70.82/3.79	C_γ_−H_γ_ in β−β (resinol) substructures (B)
B_γ_	69.68/3.52	70.55/3.46	C_γ_−H_γ_ in β−β (resinol) substructures (B)
X_2_	69.86/3.08	70.54/3.02	C_2_−H_2_ in β–d–xylopyranoside (X)
X_3_	71.66/3.19	72.26/3.14	C_3_−H_3_ in β–d–xylopyranoside (X)
X_4_	73.20/3.41	73.69/3.36	C_4_−H_4_ in β–d–xylopyranoside (X)
X_5_	61.68/3.29	63.05/3.10	C_5_−H_5_ in β–d–xylopyranoside (X)
D_α_	ND	76.2/4.84	C_α_−H_α_ in benzodioxane substructures (D)
D_β_	ND	78.6/4.10	C_β_−H_β_ in benzodioxane substructures (D)
A_α_	81.17/4.91	81.71/4.86	C_α_−H_α_ in lignocresol (A)
IC_α_	86.07/5.43	86.61/5.37	C_α_−H_α_ in phenylcoumaran substructures (IC)
S_2,6_	106.1/6.58	106.7/6.53	C_2, 6_−H_2, 6_ in syringyl units (S)
S’_2,6_	106.2/7.30	ND	C_2,6_−H_2, 6_, C(α)=O in syringyl units (S’)
G_2_	112.9/6.91	113.6/6.86	C_2_−H_2_ in guaiacyl units (G)
G_5_	114.6/6.63	115.3/6.57	C_5_−H_5_ in guaiacyl units (G)
G_6_	120.6/6.66	119.8/6.50	C_6_−H_6_ in guaiacyl units (G)
H_3,5_	116.6/6.50	117.3/6.45	C_3, 5_−H_3,5_ in *p*-hydroxyphenyl units (H)
H_2,6_	129.7/7.11	129.1/7.07	C_2, 6_−H_2,6_ in *p*-hydroxyphenyl units (H)
C_2_	ND	112.2/6.90	C_2_−H_2_ in catechyl units (C)
C_5_	ND	115.1/7.04	C_5_−H_5_ in catechyl units (C)
A_2,6_	129.8/7.04	130.2/7.02	C_2,6_−H_2,6_ in lignocresol (A)
A_3_	108.3/6.71	108.9/6.65	C_3_−H_3_ in lignocresol (A)
L_3,5_	110.2/6.90	110.8/6.84	C_3,5_−H_3,5_ in cresol (L)
*p*CA_2,6_	130.0/7.35	130.5/7.29	C_2, 6_−H_2,6_ in *p*-coumarate (*p*CA)
*p*CA_3,5_	118.0/6.76	118.7/6.71	C_3, 5_−H_3,5_ in *p*-coumarate (*p*CA)
FA_2_	113.6/7.12	114.4/7.11	C_2_−H_2_ in ferulate (FA)
FA_6_	121.8/6.87	122.3/6.82	C_6_−H_6_ in ferulate (FA)
F_α_	128.1/6.88	128.8/6.82	C_α_−H_α_ in *p*-hydroxycinnamyl alcohol (F)
F_β_	126.6/6.74	127.3/6.69	C_β_−H_β_ in *p*-hydroxycinnamyl alcohol (F)
F’_β_	124.8/6.74	125.4/6.68	C_β_−H_β_ in cinnamaldehyde end groups (F’)

δ_C_/δ_H_ (ppm) ^a^, the HSQC spectra assignments of control switchgrass lignocresol sample. δ_C_/δ_H_ (ppm) ^b^, the HSQC spectra assignments of transgenic switchgrass lignocresol sample. ND, no detected.

**Table 5:** The units and linkages proportion of switchgrass LCs.

Sample	S/%	G/%	S/G	H/%	β-*O*-4%	β-5/%	β-β/%	*p*CA/FA
Transgenic	19.70	69.46	0.28	10.84	74.51	19.34	6.14	2.56
Control	41.22	48.42	0.85	10.36	80.72	14.56	4.68	3.25
